# Study protocol: healthy urban living and ageing in place (HULAP): an international, mixed methods study examining the associations between physical activity, built and social environments for older adults the UK and Brazil

**DOI:** 10.1186/s12889-018-6018-0

**Published:** 2018-09-21

**Authors:** Geraint Ellis, Ruth F. Hunter, Adriano Akira F. Hino, Claire L. Cleland, Sara Ferguson, Brendan Murtagh, Ciro Romelio Rodriguez Anez, Sara Melo, Mark Tully, Frank Kee, Urmi Sengupta, Rodrigo Reis

**Affiliations:** 1 School of Natural and Built Environment, David Keir Building, Stranmillis Road, Belfast, BT9 5AG UK; 2 UKCRC Centre of Excellence for Public Health (NI), Queen’s University Belfast, Belfast, BT12 6BA UK; 3 Postgraduate in Health Technology, Pontifícia Universidade Católica do Paraná, Curitiba, Brazil; 4 Department of Physical Education, Federal University of Technology (UTFPR), Curitiba, Paraná Brazil; 5 Queen’s Management School, Queen’s University Belfast, Belfast, BT9 5EE UK; 6 Prevention Research Center, Brown School, Washington University in St. Louis, St Louis, USA; 7 Research Group in Physical Actity and Quality of Life (GPAQ), Pontifícia Universidade Católica do Paraná, Curitiba, Brazil; 8 Postgraduate Program in Urban Management (PPGTU), Pontifícia Universidade Católica do Paraná, Curitiba, Brazil

**Keywords:** Protocol, Older adults, Healthy urban living, Ageing in place, Built environment, Social environment, Physical activity knowledge exchange

## Abstract

**Background:**

The ability to ‘age in place’ is dependent on a range of inter-personal, social and built environment attributes, with the latter being a key area for potential intervention. There is an emerging body of evidence that indicates the type of built environment features that may best support age friendly communities, but there is a need to expand and consolidate this, while generating a better understanding of how on how research findings can be most effectively be translated in to policy and practice.

**Methods:**

The study is based on two case study cities, Curtiba (Brazil) and Belfast (UK), which have highly contrasting physical, social and policy environments. The study deploys a mix methods approach, mirrored in each city. This includes the recruitment of 300 participants in each city to wear GPS and accelerometers, a survey capturing physical functioning and other personal attributes, as well as their perception of their local environment using NEWS-A. The study will also measure the built environments of the cities using GIS and develop a tool for auditing the routes used by participants around their neighbourhoods. The study seeks to comparatively map the policy actors and resources involved in healthy ageing in the two cities through interviews, focus groups and discourse analysis. Finally, the study has a significant knowledge exchange component, including the development of a tool to assess the capacities of both researchers and research users to maximise the impact of the research findings.

**Discussion:**

The HULAP study has been designed and implemented by a multi-disciplinary team and integrates differing methodologies to purposefully impact on policy and practice on healthy ageing in high and low-middle income countries. It has particular strengths in its combination of objective and self-reported measures using validated tools and the integration of GPS, accelerometer and GIS data to provide a robust assessment of ‘spatial energetics’. The strong knowledge exchange strand means that the study is expected to also contribute to our understanding of how to maximise research impact in this field and create effective evidence for linking older adult’s physical activity with the social, built and policy environments.

## Background

### Background and rationale

The number of older adults^1^ is increasing worldwide, both absolutely and as a proportion of the global population [1–5]. The United Nations have stated that if the world’s population continues its current trajectory, those aged 80 years and older will increase threefold to 392 million over the next 30 years [6]. This shift in demographic profile has had, and will continue to have, substantial implications for a wide range of policy fields including transport, planning, housing and most notably health and social care [2, 6–8]. While longevity is something to be celebrated, a rapidly ageing population will place increased strain on public health services and budgets, as older adults live longer with non-communicable diseases, disabilities and potentially, poorer quality of life [9–11]. Although this is being experienced differently in each global region, such a profound demographic trend is likely to cause significant social transformation and it is imperative that this is prepared for throughout the world [4]. A key concept here is that of ‘healthy ageing’, regarded as being “the process of optimising opportunities for physical, social and mental health to enable older people to take an active part in society without discrimination and to enjoy an independent and good quality of life” [12]. This is not just about ensuring older adults live in the absence of illness, but that they have the ability to fulfil their full capacity and do what they value. This is such a vital global challenge that the WHO has declared that 2020–2030 will be the ‘Decade of Healthy Ageing’ [13].

Healthy ageing is not just determined by the intrinsic capacity of the individual, but also due to the social, economic, political and built environments in which they live [13, 14], so there is much interest in how we can build age-friendly communities that support older adults’ health, functioning, independence and physical activity [15–17] . Cities have a key role in enabling older people to live longer and healthier lives while fostering more productive societies. They therefore need to find sustainable models that leave nobody behind for basic health and social services, education, decent jobs, housing, transportation, security and safety. An important objective is to help older adults to ‘age in place’ [18] i.e. “remaining living in the community, with some level of independence, rather than in residential care” [19], with an aspiration to stay in their own home as long as possible, maintain established social networks and in so doing continue to contribute to local economies and rely less on health and social services [20]. A key determinant of being able to ‘age in place’ is an enduring capacity of an individual to engage in physical activity, which both supports and maintains independent living. Indeed, an adequate level of physical activity is a key factor in preventing population wide non-communicable diseases and is associated with a wide range of health benefits and a 47% reduction in mortality [9, 21]. A supportive built environment has a significant long and short term influence on this [22–24] so there is a need to identify the built environment features that best support ‘ageing in place’ and understand the ways in which this can be translated into actionable interventions and then to ‘scale up … programs, policies and actions’ (p. 10) to address declining levels of physical activity [25].

There is already a significant body of research that demonstrates the features and form of the built environment which influences levels of physical activity amongst all ages and social groups of the population [26, 27]. It has been suggested that built environment attributes such as connectivity, accessibility, land use mix, residential density and environmental quality can influence levels of physical activity; and more detailed urban design features can have a key role in enhancing physical activity by improving the pedestrian experience [28–30]. There are important empirical and conceptual insights into how we might design interventions in the built environment that promote a healthier approach to urban planning and increase levels of physical activity for all age groups. However conventional ‘walkability’ is based on assumptions around able-bodied walking abilities and does not reflect the diversity and ages of the population as a whole [31]. Indeed, to date the impact of both the built and social environments have been overlooked in terms of how they influence older adults’ physical activity.

‘Older adults’ are a highly differentiated group, they will have specific, and complex relationships with the intra-personal, social and built environments in which they live, with the latter becoming increasingly important as daily activities contract to immediate surroundings as mobility and functional impairment increases with age [32, 33]. Recent systematic reviews of the emerging research on built environment influences on older adults’ physical activity, walking and active travel [34–37] have noted how pedestrian infrastructure, safety, lighting, access to local services, green open space recreational facilities and other features of walkability (density, land use mix etc) appear positively associated with walking and physical activity whilst aesthetically disruptive features such as litter, vandalism and decay appear to be negatively associated with walking. These reviews also highlight some key challenges of research in this field, including the need to conduct studies in countries other than the United States and the need to combine validated and standardised objective *and* self-reported physical activity, across different domains [36]. Moran et al. [34] suggest that future quantitative studies should not only account for the presence of environmental attributes, but also their quality and that there should be a preference for inter-disciplinary studies that combine spatial analysis with health data. Barnett et al. [37] also highlight the need to unpack the mechanisms between domain-specific physical activity and built environment features and how issues such as crime or personal safety issues relate to total health enhancing physical activity, while emphasising the need for multi-country pooling of data using valid comparable measures. Cerin et al. [35] further emphasise the need to understand how particular behaviours (physical activity and sedentary) relate to specific built environment characteristics and the need to better understand the complex interactions of the social, built and political environments with socio-demographic, health or psychosocial factors, highlighting how the use of GPS can aid in overcoming some of these constraints. Cerin et al. [37] also draw attention to some of the weaknesses in the research in this field, with sampling bias and a range of unacceptable analytical practices, such as a tendency to transform continuous outcomes and exposures into categories, being particularly common. Therefore, while there is an emerging body of evidence that indicates the type of built environment features that may best support ageing in place, there is still a need to expand and consolidate the insights already developed through well-designed, cross-country comparative studies using a mix of methods, including validated objective and self-reported measures of domain specific physical activity, coupled with standardised spatial data relating to the built environment.

However, even where robust data exists on built environment influences on physical activity, there are still significant barriers in the knowledge exchange (or translation) mechanisms that can help guide policy-makers and other practitioners to design appropriate interventions, and conversely, informing researchers of the type of knowledge best needed to inform policy and practice [38]. Built environment/health research faces specific difficulties of translation into policy because the environment tends to be treated as a ‘black box’ of multiple elements, and the lack of clarity of which myriad of physical attributes really make a difference. Furthermore, there has been a tendency to capture subjective measures in a way that they cannot be easily translated into policy interventions or that receptive agencies usually only have control over a limited number of relevant factors [22]. Indeed, knowledge exchange^2^ is a fluid and dynamic process that needs to be considered in relation to its specific practice settings [30] and is often side-lined by both researchers and practitioners. Closely related to the tradition of ‘dissemination and implementation’ research in public health [38], an emphasis on the exchange of knowledge has implications for not just post-research dissemination of findings but should also involve researcher/research-user dialogue throughout the research process. Although this is an issue that faces all public health research, effective translation of the type of evidence that would support interventions for healthy ageing in place faces a particular set of challenges and involves a very diverse spectrum of research fields, policy actors and statutory agencies including frontline health and social services, third sector advocacy agencies and authorities responsible for transport, planning, development and the maintenance of the built environment. The different constellations of such actors will also vary enormously depending on geographic and institutional context, so that generalised models and guidelines for knowledge exchange are of limited value. Because of this, the process of translating evidence into policy and practice should become embedded into every stage of the research process [39] and preferably, involve those who are best placed to use resulting evidence from research themselves at the earliest stages.

Therefore, in the context of an increasingly ageing global population there is a need to take forward research that both improves our understanding of how best to design neighbourhoods that can support older adults’ health, functioning, independence and physical activity, as well as producing evidence in a way that can more directly speak to the requirements of key decision-makers and other stakeholders. As noted above, such research also should be developed in a context that reflects a wide range of environmental variability of urban environments and one which can respond to the diverse cultural and demographic contexts of ageing. Western Europe provides one distinct setting for ageing in place, having urbanised and industrialised, de-industrialised and then witnessed the upward trend of an ageing society. This contrasts with regions in the Global South, many of which are facing immense processes of urbanisation and undergoing profound shifts in demographic change at the same time. For example, the same demographic process of low fertility and high longevity that took place in a century in Western Europe is unfolding in Brazil in just two decades. Brazil currently had in 2015 a median age of 31.3 years, a proportion of elderly (over 60) people in the population of 11.9%, and 16.4 million people aged 65 and over [4] . However, by 2050, there will be 53.3 million people aged 65 and over, or 22.9% of the population [4]. In the UK, the median age is 40, with 20.1 million people over 60 in 2014, projected to increase to 31.8 million by 2039 [40]). There will be particularly steep increases in the number of older old adults, with an increase of the over 75 s of nearly 90% during this time so by 2039 1 in 12 of the UK population will be 80 or over. If these demographic profiles are also located with the very diverse urban environments and urbanisation processes occurring in the UK and Brazil, we see a startling contrast of opportunities and constraints for address the in challenge of healthy ageing in place.

The trends in global ageing, the aspiration to experience healthy ‘ageing in place’ combined with the potential influences of the built environment led to the design of the three-year *Healthy Urban Living and Ageing in Place: Physical Activity, Built Environment & Knowledge Exchange in Brazilian Cities* (HULAP) [41] project which focuses on a Brazil-UK comparison on the physical activity behaviour of older adults, the built environments in which they live, the efficacy of the wider policy terrain for ‘ageing in place’ and the mechanisms for best translating emerging findings into policy and practice. The project is focused in two case study cities: Belfast in the United Kingdom (UK) and Curitiba in Brazil. These are selected for the particular characteristics rather than being ‘representative’ of their respective global regions; for example, Curtiba is renowned for its innovative approach to sustainable transport [42] while Belfast shows high car dependency, even by European standards [43]. The project is being undertaken through an inter-disciplinary consortium of researchers from Queen’s University Belfast, Pontifical Catholic University of Parana, Washington University in St Louis and the Federal University of Technology, Parana, drawing on expertise from public health, spatial planning, geography, urban design and management.

### Aims and objectives

The overall aim of the HULAP Project is to: ‘*enhance the conceptual and empirical understanding of the influence of built, social, political and policy environments on physical activity and sedentary behaviours of older adults, and to develop evidence and policy tools for increasing physical activity and well-being of older adults in the United Kingdom and Brazil through interventions, enhanced policy effectiveness and improved institutional collaboration’*.

This is further specified through objectives that relate to three identified themes, seven Work Packages (see below) and the following research questions:

*Theme 1: Urban Design, Planning, Housing and Infrastructure*.

1. How does the objectively measured walkability vary within and between Brazilian and UK cities?
2. How effective is the objective Walkability Index in capturing the associations for measuring effectiveness for older adults and the urban context of Brazil and can these be improved by using other built environment attributes?

*Theme 2: Health Inequalities and Justice*

3. What are the patterns of physical activity and sedentary behaviour amongst a sample of older adults in Brazil and the UK?
4. What are the associations and moderating effects of objective and perceived environment measures with physical functioning, BMI and other specific age-related attributes of older adults in Brazil and the UK?

*Theme 3: Leadership, Governance and Institutions*

5. What are the wider economic and social contexts for healthy ageing in the UK and Brazil?
6. What are the key policy actors, institutions, programmes, staff and resources involved in ageing and walkable environments in Brazil and the UK?
7. How effective are the evidence-policy interactions and opportunities for knowledge exchange around healthy ageing in Brazil and the UK?
8. What are the main opportunities for increasing physical activity for older people in Brazilian and UK Cities?
9. What projects, programmes and areas of institutional reform need to be in place to best enhance the opportunities for healthy ‘ageing in place’ in Brazilian and UK cities?

## Methods/design

The HULAP Project is an international multi-disciplinary collaborative, mixed methods study, involving two case study cities, Belfast (UK) and Curitiba (Brazil) which allow for a comparative evaluation of the varied social and built environments between these two countries based on a sample of high and low walkability and income neighbourhoods in each city. Both countries will use quantitative (objective physical activity and sedentary behaviour measurement, GPS tracking, GIS, built environment audit and a survey) and qualitative (focus groups, interviews) methods with samples of older adults; which will then be complemented by literature reviews, qualitative interviews with stakeholders, policy mapping, the development of walkability tools and neighbourhood audits. When the data gathering process is complete it will enable the research team to develop strategies to influence research, policy and practice; consequently, promoting healthy urban ageing.

**Fig. 1:**
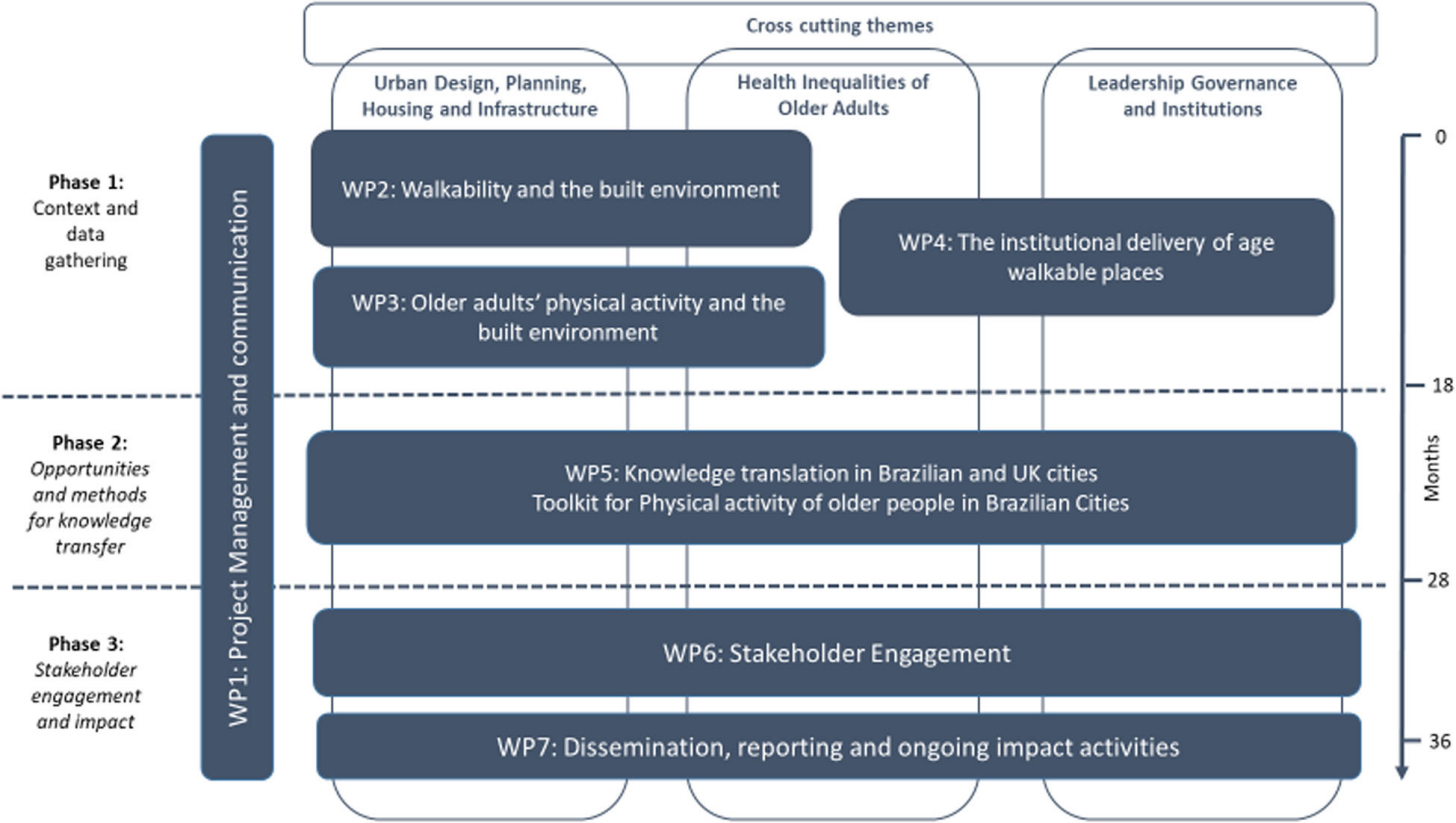
Work package structure of the HULAP project

The Project has been structured into three phases relating to: 1) context comprehension and data gathering; 2) exploring opportunities and means for knowledge exchange; 3) Stakeholder engagement and impact. The project is also broken down into 7 Work Packages (WPs) described below and shown in Fig. 1. The methods are described in the next section, according to the WP structure.

### Work package 1: Project management and communication

**Fig. 2:**
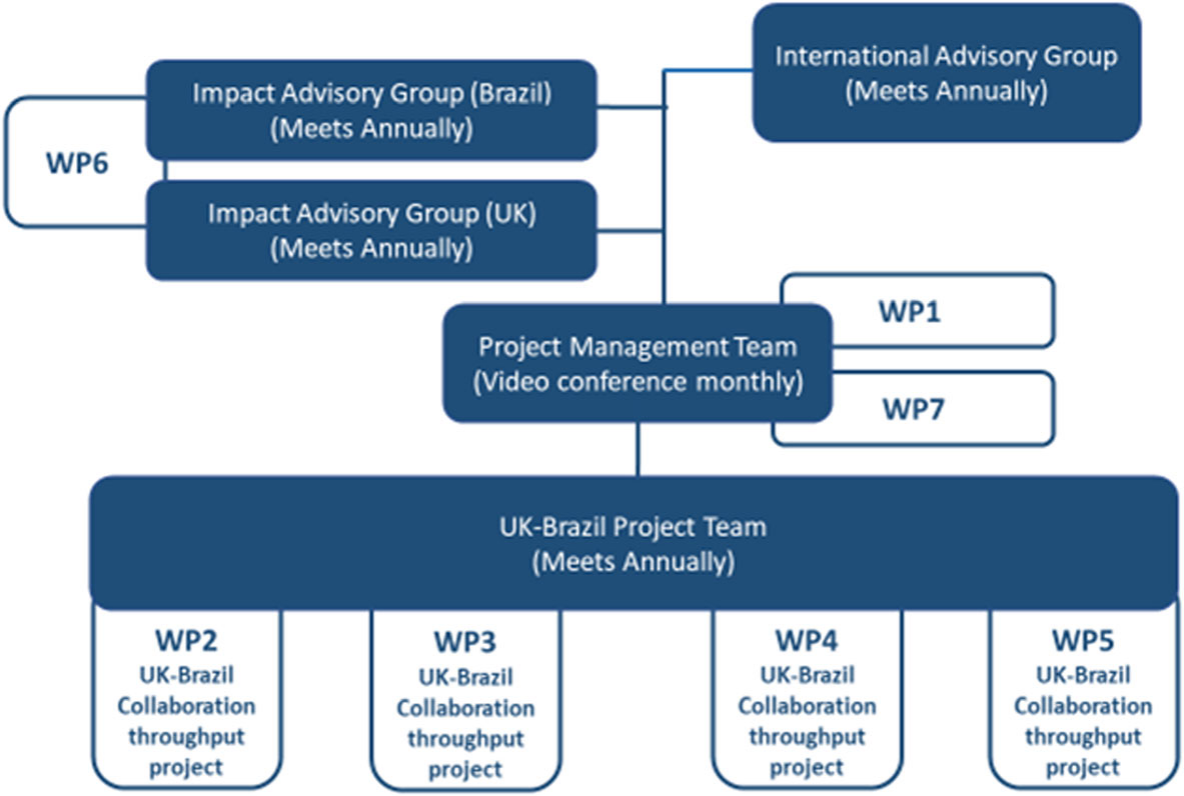
HULAP Project Management arrangements

The purpose of this WP is to ensure effective project management and communication with project team and other partners, delivery of all outputs and ultimate achievement of the research aims, under the joint responsibility of the UK and Brazil lead researchers. Arrangements for the management of the project are shown in Fig. 2.

### Work package 2: Walkability and the built environment

The purpose of this WP is to capture the built environment attributes of Belfast (UK) and Curitiba (Brazil) that may support or impede physical activity of older adults and develop tools for specifically measuring ‘older adults’ walkability’ in Brazilian cities. The key deliverables are three datasets: walkability indices for Belfast/Curitiba; systematic review and focus group data on built environmental attributes of specific relevance to older adult’s physical activity and mobility; an audit of built environment used by older adult participants.

#### *WP2 Methods:* This WP has four strands of activity

2.1 Establish objectively measured walkability indices (based on density, land use mix, connectivity, [44] for the cities of Curitiba and Belfast, and specifically for the 400 m/500 m/1000 m hinterlands of the older adult participants in WP3 (UK *n* = 300, Brazil *n* = 300) drawing on previous work undertaken by the researchers in the UK and Brazil [45, 46].
2.2 In accordance with the existing IPEN approach, a series of built environment attributes will be incorporated into the ‘IPEN GIS template and variable naming conventions’ document will be modelled using GIS methods. This work package also aims to identify and model additional attributes of the built environment that may have a specific influence on physical activity of older adults in Belfast and Curitiba. This will be undertaken through an international systematic review of existing literature on healthy ageing and older adults’ physical activity to identify built environment attributes that could promote or impede older adults’ physical activity and sedentary behaviour in both high and low-middle income countries. The findings from the review will then be validated through focus groups with older adults in both the UK and Brazil. Focus groups will be conducted in areas of varying walkability and socio-economic status, and subsequent data will be analysed using a thematic analysis approach. Focus group findings will cross-checked with the findings from the systematic review in order to determine if any additional built environment attributes should be included within additional GIS and streetscape audit analyses.
2.3 An extensive list of attributes (identified in strand two above) which will then be used to modify the existing Microscale Audit of Pedestrian Streetscapes (MAPS) Global tool [47, 48], which can be used to audit the micro-features of the built environment for influence on physical activity of older adults. The routes most commonly-used by qualifying participants (i.e. of the n.300 from each city, those with valid walking trips recorded in a buffer around their home) will be identified though analysis of the GPS data collected as part of WP3 (see below). The ‘most common route’ used (for active travel or recreation) around their home will be identified and then an alternative route (‘least common route’) paired with this and compared. The first 400 m of these routes will then be audited using the MAPS Global protocol, adjusted to collect additional variables identified in previous focus groups as having specific relevance to older adult’s mobility. The associations of built environment features will then be analysed against the physical activity behaviour recorded in WP3, with a view to testing the viability of a specialised ‘MAPS Seniors’ tool depended on the strength of association of added built environment attributes.
2.4 The data collected above will then be synthesised and analysed to draw out comparative insights between built environments in the UK and Brazil and combined with the outcomes of WP3 to develop a regression model to identify facilitating/impeding built environment features for physical activity of older adults and recommend both future auditing processes and potential built environment interventions.

### Work package 3: Older adults’ physical activity, sedentary behaviour and the built and social environment

The purpose of this WP is to understand the type, intensity and location of older adults’ physical activity and sedentary time using the integration of objective measurement techniques in the UK and Brazil. It also collects data on self-reported physical activity, sedentary behaviour and perceptions of the neighbourhoods in which participants live. This facilitates an analysis of the associations between objective and environment measures with physical functioning, BMI and specific age-related attributes.

The study, and this WP, is guided by a specific multi-level ecological model. At least 600 older adults aged 60 years and above, will participate, 300 from each city. In both countries, older adults will be recruited from neighbourhoods (Administrative Units) selected to vary in walkability (identified through GIS analysis in WP2) and socioeconomic status, with simultaneous recruitment in each study “quadrant”: 1) Low socio-economic status/low walkability; 2) low socio-economic status/high walkability; 3) high socio-economic status/low walkability; and 4) high socio-economic status and high walkability. Analyses will adjust for multi-level clustering and individual demographics. The mode of recruitment varies by country: in Brazil, participants living in eligible residential areas will be identified using systematic “door-to-door” recruitment in identified census tracts. In Northern Ireland, participants living in eligible neighbourhoods will be identified from the NICOLA study [49]. NICOLA is Northern Ireland’s long-term study of ageing involving 8500 men and women aged 50 years and over randomly selected from the community and representative of the population.

#### *WP3 Methods.* This WP has two main strands of activity

3.1 Older adults aged 60 years and older, in the UK (n.300) and Brazil (n.300) will wear accelerometers (Actigraph GT3X) and GPS (Qstarz BT-Q1000XT) for 7 days. This will provide researchers with a measure of not only what physical activity participants do in terms of minutes of moderate-to-vigorous physical activity and sedentary behaviour, but it will also provide researchers with the answer of where participants perform physical activity or where they are sedentary.
3.2 The same participants will be asked to complete a validated and systematically adapted survey in order to record their perceptions of local built and social environment attributes (NEWS-A [50]), psychosocial and demographic variables, and additional outcomes (physical functioning, health conditions). These measures will supplement the accelerometer and GPS data and allow analyses of conceptually-matched environmental features with specific physical activity and sedentary behaviours.

### Work package 4: The institutional delivery of age walkable places

The purpose of this WP is to comparatively map the policy actors, organisations, programmes, staff, skills and resources involved in ageing and walkable environments in the UK and Brazil, in order to understand the wider institutional capacities and influence on delivering healthy aging in place interventions. The WP also seeks to identify examples of innovative projects and programmes in Belfast and Curitiba in order to learn from how the wider institutional landscape can encourage or constrain responses to the challenges of healthy ageing.

#### *WP4 Methods*. This WP has three main strands

4.1 The first strand involves a process of critical policy analysis, conducted through a discourse analysis using text based analytical software to identify the critical narratives in key documents (focused on planning, housing and transport) related to healthy ageing and the built environment in Belfast and Curitiba. These will be mapped and compared to evaluate how ageing in place is understood, and how these are expressed in arenas such as spatial planning, transport and social care policy.
4.2 There will also be a series of semi-structured policy and practice based interviews, with key stakeholders from central and local government, private providers and NGOs (*n* = 25 in both the UK and Brazil) aimed at evaluating: how ageing in place is understood by policy makers and practitioners; what proposals or programmes exist; how they analyse and evaluate conditions, anticipate risk and manage contingencies; what are the institutional, cultural, knowledge or professional obstacles to the development of more progressive policies; and examine the models and practices that have been attempted and with what degree of success. This will again use discourse analysis to gain a more comprehensive and multi-sectoral appreciation of the everyday policy realities of healthy ageing and walking in the built environment specifically.
4.3 The final strand to WP4 will review and compare existing *innovative practices* (*n* = 10 in each city) for ‘ageing in place’ related projects and programmes already operating in the two case study cities. These will include community-based responses to loneliness, community integration and social enterprise development and compare a range of state, private and voluntary responses in Brazil and the UK to the spatial implications of an ageing society.

### Work package 5: Knowledge translation in Brazilian and UK cities

The purpose of this WP is to identify appropriate knowledge exchange and translation mechanisms for enhancing the impact of the project in Belfast and Curitiba around healthy ageing to make recommendations for accommodating the needs of older adults in the planning, social and health services in UK and Brazilian cities. The WP will also seek to prepare key guidance (design tool kits etc) for policy-makers in Curitiba and Belfast on how to support ageing in place through built environment interventions. The key deliverables are a self-assessment tool for knowledge translation aimed at both researchers and potential research users; a dataset resulting from surveys using the self-assessment tool, supplemented by interviews and focus groups.

#### *WP5 Methods*. This WP involves four key strands

5.1 The first strand involves a review of knowledge translation processes and tools, which will involve a literature review, gathering examples of good practice and a review of existing examples of good practice in the UK and Brazil for knowledge exchange in the area of healthy ageing.
5.2 The WP will undertake focus groups (one each in UK and Brazil, *n* = 2) and interviews (eight in the UK, eight in Brazil, *n* = 16) with key stakeholders in fields related to ageing in place (built environment, mobility, social care etc.) to ascertain current challenges and ways to improve knowledge exchange in this area.
5.3 The third strand involves the review of existing self-assessment tools for knowledge translation (including SATORI developed by Gholami et al. [51] and the *Self-Assessment Tool* developed by the Canadian Foundation for Healthcare Improvement [52]. This review will be used to develop a tool for assessing capacities, resources, and other issues crucial to knowledge exchange on healthy ageing in place in Belfast and Curitiba.
5.4 The final strand of this WP will be to generate outputs that best translate the findings of the research (particularly that from WP2 and WP3) using media and formats that have been identified as being the most effective for the stakeholders identified in WP4, using by the self-assessment tool developed here combined with the interviews and focus groups. This could potentially take the form of infographics, briefing papers or design tools.

### Work package 6: Stakeholder engagement

The purpose of this WP is to give older people, regulatory agencies and other stakeholders in Belfast and Curitiba a voice in helping shape the research and policy agenda and directly advising on the research approach, its analysis and outputs. The key deliverables from this work package are advisory reports, communication plans and forums that bring together key stakeholders, both within and across the two case study cities. Indeed, the research design being described here, was developed through consultation with a number of these stakeholders and to maintain this level of involvement, an intermediary Impact Advisor will be engaged in each city to coordinate the WP activities. In Belfast this is WHO Belfast Healthy Cities [53] and in Curitiba an Advisory Team composed by key participants mapped during the WP4 from organisations as the City Urban Planning Institute, Municipal Council for Older Adults and other key local agencies and organization involved with local policies and programs on active ageing. These intermediaries will facilitate wider Impact Advisory Groups, in both Brazil and Curitiba, with opportunities for these two groups to themselves interact – once in Brazil; and once in the UK. The aim of these meetings will be to share experience, expertise and local models of good practice on active ageing and explore opportunities for implementing research recommendations in order to maximise impact of the project. The Impact Advisory Groups will also be given the specific tasks of peer reviewing several outputs from the project, including an older adults’ friending walkability toolkit.

### Work package 7: Dissemination, reporting and ongoing impact activities

The purpose of the final work package is to ensure the findings from each of the work packages are synthesised and research findings disseminated to key and diverse audiences comprising of academic researchers, policy makers, advocacy groups, older adults, and other research users. The synthesis of the various work packages will take place through integration of the data sets identified above and guided by the main policy driven questions that emerge from the Impact Advisory Groups. This will guide the publication strategy of academic papers across a variety of disciplinary fields, develop briefing papers for key findings that emerge from the project and ensure all data sets are adequately catalogued and archived in accessible repositories.

## Ethics and dissemination

The HULAP study has secured ethical approval from both Queen’s University Belfast (QUB) and Pontifícia Universidade Católica do Paraná, in compliance with the ESRC Framework for Research Ethics [54]. In the case of QUB, approval has been granted by the School of Medicine and Biological Sciences in relation to the sub-sample of participants from the NICOLA study (for collection of survey, accelerometer and GPS data), and from the Faculty of Engineering and Physical Sciences in relation to other data collection, including interviews and survey of policy stakeholders. Informed consent will be secured from all participants by project researchers, guided by specific guidance on Ethical Practice in HULAP that will, inter alia, help to identify vulnerable situations and groups, the need for sensitivity in handling specific issues, safety and security of researchers during fieldwork and how to maintain confidentiality.

Data collected as part of the project will be held in accordance with the ESRC Research Data Policy [55] and universities’ policies on management of physical research data and on working with electronic data. Standard Operating Procedures (SOPs) will be drawn up for the backup, storage and security of research data, with access limited to designated staff during the life of the project. On completion of the project, all data will be lodged with university data repository and will make the data available and for re-use according to FAIR data principles (findable, accessible, interoperable, re-usable).

Dissemination of project findings will be made via academic papers published in peer reviewed journals and through working papers and policy briefs that will be disseminated directly to stakeholders and via the project website. Other published outputs will be developed according to the needs of stakeholders, as identified through the knowledge exchange component of the project. Individual participants will also be provided with a lay-summary of key project findings.

## Discussion

The HULAP Project is an international multi-disciplinary collaborative study between the UK and Brazil, which seeks to enhance the empirical understanding of the influence of built and social environment on physical activity of older adults and develop a conceptual understanding of the forms of urban governance that support healthy ageing. It also aims to develop evidence and policy tools for increasing physical activity and well-being for ‘ageing in place’. The strengths of the study include the combination of objective and self-reported measures, using validated tools. The combination of GPS, accelerometery, GIS and ‘micro-audit’ of the built environment allows temporal activity in specific physical activity domains to be associated with high resolution built environment features to provide a robust assessment of ‘spatial energetics’ [56]. It therefore seeks to overcome many of the common limitations facing studies in this field [35, 37]. Other key features of the project include the involvement of researchers from a wide and diverse range of disciplinary backgrounds (including spatial planning, public health, geography, management and specialist physical activity researchers) in a dynamic and integrated research team, bringing complementary perspectives to the challenges of ageing in place. In particular the project is relatively unusual in terms of the level of integration of knowledge exchange involved in the project, including a specific WP aimed at understanding the needs and capacities of those working in relevant policy sectors, the involvement of impact intermediaries to advise on the project as it is implemented and the focus on specific impact related outputs that will emerge from the project. As such it is expected that the project will contribute as much to the understanding of knowledge exchange processes in this field as it will to the hard body of evidence linking older adult’s activity with the physical built environment.

The potential limitations of the study include the fact that a single city from each country cannot be regarded as being representative of their country or global region. Indeed, each city has specific characterises that make them of particular interest and which offer a particular wide range of environmental attributes to strengthen the analysis of built environment/physical activity associations.

## Abbreviations

ESRC: Economic and Social Research Council
GIS: Geographic information system
GPS: Global position system
HULAP: Healthy urban living and living in place study
IPEN: International physical activity and environment network
MAPS: Microscale audit of pedestrian streetscapes NEWS-A: Neighbourhood Environment Walkability Scale (Abbreviated)
NGOs: Non-governmental organisations
PA: Physical activity
SB: Sedentary behaviour
SOPs: Standard operating procedures
WHO: World Health Organisation
WP: Work Package

## Footnotes

1 Here regarded as being 60 years or older.

2 For the purposes of this discussion we regard ‘knowledge exchange’ as the process that brings academics together with users of research and other stakeholders to exchange ideas, evidence and expertise and that this can include complex and diverse activities which can deliver economic and societal benefits over varying timescales.

## References

[1] Garin N, Olaya B, Moneta MV, Miret M, Lobo A, Ayuso-Mateos JL (2014). Impact of multimorbidity on disability and quality of life in the Spanish older population. PLoS One.

[2] Partridge L (2014). Intervening in ageing to prevent the diseases of ageing. Trends Endocrinol Metab.

[3] Mathers CD, Stevens GA, Boerma T, White RA, Tobias MI (2015). Causes of international increases in older age life expectancy. Lancet.

[4] United Nations (2015). Department of Economic and Social Affairs, Population Division, World Population Ageing.

[5] Witard OC, McGlory C, Hamilton DL (2016). Growing older with health and vitality: a nexus of physical activity, exercise and nutrition. Biogerontology.

[6] United Nations (2013). World Population Ageing.

[7] World Health Organization (2002). Active aging: a policy framework.

[8] World Health Organization (2015). World report on ageing and health.

[9] Gebel K (2015). Effect of moderate to vigorous physical activity on all-cause mortality in middle-aged and older Australians. JAMA Intern Med.

[10] Jin K, Simpkins JW, Ji X, Leis M, Stambler I (2015). The critical need to promote research of aging and aging-related diseases to improve health and longevity of the elderly population. Ageing Dis.

[11] Lehnert T, Heider D, Leicht H (2011). Review: health care utilization and costs of elderly persons with multiple chronic conditions. Med Care Res Rev.

[12] Agren G, Berensson K. Healthy ageing: a challenge for Europe: Swedish National Institute of Public Health; 2006.

[13] World Health Organization (2017). *10 Priorities:* Towards a decade of healthy ageing.

[14] Suzman R, Beard JR, Boerma T, Chatterji S (2015). Health in an ageing world—what do we know?. Lancet.

[15] Fitzgerald KG, Caro FG (2014). An overview of age-friendly cities and communities around the world. J Ageing Soc Policy.

[16] Jeste DV, Blazer DG, Buckwalter KC, Cassidy KLK, Fishman L, Gwyther LP, Levin SM, Phillipson C, Rao RR, Schmeding E, Vega WA (2016). Age-friendly communities initiative: public health approach to promoting successful aging. Am J Geriatr Psychiatry.

[17] Steels S (2015). Key characteristics of age-friendly cities and communities: a review. Cities.

[18] Wiles JL, Leibing A, Guberman N, Reeve J, Allen RE (2012). The meaning of “aging in place” to older people. The Gerontologist.

[19] Davey J, Nana G, de Joux V, Arcus M (2004). Accommodation options for older people in Aotearoa/New Zealand.

[20] Cheek J (2007). From retirement village to residential aged care. Health Soc Care Community.

[21] Physical Activity Guidelines Advisory Committee (2018). Physical activity guidelines advisory committee report.

[22] Burton EJ, Mitchell L, Stride C (2011). Good places for ageing in place: development of objective built environment measures for investigating links with older people’s wellbeing. BMC Public Health.

[23] Frank LD, Kerr J, Rosenberg D, King A (2010). Healthy aging and where you live: community design relationships with physical activity and body weight in older Americans. J Phys Act Health.

[24] Haselwandter EM, Corcoran MP, Folta SC, Hyatt R, Fenton M, Nelson ME (2015). The built environment, physical activity, and aging in the United States: a state of the science review. J Aging Phys Act.

[25] Schmidt MI (2011). Chronic non-communicable diseases in Brazil. Lancet.

[26] Ding D, Gebel K (2012). Built environment, physical activity, and obesity. Health Place.

[27] Sallis JF, Cerin E, Conway TL, Adams MA, Frank LD, Pratt M, Salvo D, Schipperijn J, Smith G, Cain KL, Davey R (2016). Physical activity in relation to urban environments in 14 cities worldwide: a cross-sectional study. Lancet.

[28] Adkins A (2012). Unpacking walkability: testing the influence of Urban Design features on perceptions of walking environment attractiveness. J Urban Des.

[29] Sallis JF, Floyd MF, Rodríguez DA, Saelens BE (2012). Role of built environments in physical activity, obesity, and cardiovascular disease. Circulation.

[30] Barnett DW, Barnett A, Nathan A, Van Cauwenberg J, Cerin E (2017). Council on, E, & physical activity – older adults working, built environmental correlates of older adults' total physical activity and walking: a systematic review and meta-analysis. Int J Behav Nutr Phys Act.

[31] Stafford L, Baldwin C (2018). Planning walkable neighborhoods: are we overlooking diversity in abilities and ages?. J Plan Lit.

[32] Chaudhury H, Mahmood A, Michael YL, Campo M, Hay K (2012). The influence of neighborhood residential density, physical and social environments on older adults' physical activity: an exploratory study in two metropolitan areas. J Ageing Stud.

[33] Carlson JA, Sallis JF, Conway TL (2012). Interactions between psychosocial and built environment factors in explaining older adults’ physical activity. Prev Med (Baltim).

[34] Moran M, Van Cauwenberg J, Hercky-Linnewiel R, Cerin E, Deforche B, Plaut P (2014). Understanding the relationships between the physical environment and physical activity in older adults: a systematic review of qualitative studies. Int J Behav Nutr Phys Act.

[35] Cerin E, Nathan A, Van Cauwenberg J, Barnett DW, Barnett A (2017). The neighbourhood physical environment and active travel in older adults: a systematic review and meta-analysis. Int J Behav Nutr Phys Act.

[36] Van Cauwenberg J, De Bourdeaudhuij I, De Meester F, Van Dyck D, Salmon J, Clarys P, Deforche B (2011). Relationship between the physical environment and physical activity in older adults: a systematic review. Health Place.

[37] Barnett DW, Barnett A, Nathan A, Van Cauwenberg J, Cerin E (2017). Built environmental correlates of older adults’ total physical activity and walking: a systematic review and meta-analysis. Int J Behav Nutr Phys Act.

[38] Brownson RC, Colditz GA, Proctor EK (2012). Dissemination and implementation research in health: translating science to practice..

[39] Neta G, Glasgow RE, Carpenter CR, Grimshaw JM, Rabin BA, Fernandez ME, Brownson RC (2015). A framework for enhancing the value of research for dissemination and implementation. Am J Public Health.

[40] Office for National Statistics (UK) (2014). National Population Projections: 2014-based Statistical.

[41] Healthy Urban Living and Ageing in Place Project: https://www.qub.ac.uk/research-centres/TheInstituteofSpatialandEnvironmentalPlanning/Impact/CurrentResearchProjects/HealthyUrbanLivingAgeinginplacePhysicalActivityBuiltEnvironmentKnowledgeExchangeinBrazilianCitiesHULAP/. Accessed 18 Apr 2018.

[42] Duarte F, Ultramari C (2011). Making public transport and housing match: accomplishments and failures of Curitba’s BRT. J Urban Plann Dev.

[43] McEldowney M, Ryley T, Scott M, Smyth A (2017). Policy agenda for the Belfast metropolitan area?. Renewing Urban Communities: Environment, Citizenship and Sustainability in Ireland.

[44] Frank LD, Sallis JF, Saelens BE, Leary L, Cain K, Conway TL, Hess PM (2016). The development of a walkability index: application to the neighborhood quality of life study. Br J Sports Med.

[45] Ellis G, Hunter R, Tully MA, Donnelly M, Kelleher L, Kee F (2016). Connectivity and physical activity: using footpath networks to measure the walkability of built environments. Environ Plann B Plann Des.

[46] Reis RS, Hino AAF, Rech CR, Kerr J, Hallal PC (2013). Walkability and physical activity: findings from Curitiba, Brazil. Am J Prev Med.

[47] Cain KL, Millstein RA, Sallis JF, Conway TL, Gavand KA, Frank LD, Saelens BE, Geremia CM, Chapman J, Adams MA, Glanz K (2014). Contribution of streetscape audits to explanation of physical activity in four age groups based on the microscale audit of pedestrian streetscapes (MAPS). Soc Sci Med.

[48] Sallis JF, Cain KL, Conway TL, Gavand KA, Millstein RA, Geremia CM (2015). Is Your Neighborhood Designed to Support Physical Activity?. A Brief Streetscape Audit Tool. Prev Chronic Dis..

[49] NICOLA study: https://www.qub.ac.uk/sites/NICOLA/ Accessed 05–04-18.

[50] Cerin E (2009). Cross-validation of the factorial structure of the neighborhood environment walkability scale (NEWS) and its abbreviated form (NEWS-A). Int J Behav Nutr Phys Act.

[51] Gholami J, Majdzadeh R, Nedjat S, Nedjat S, Maleki K, Ashoorkhani M, Yazdizadeh B (2011). How should we assess knowledge translation in research organizations; designing a knowledge translation self-assessment tool for research institutes (SATORI). Health Res Policy Syst.

[52] Canadian Foundation for Healthcare Improvement (2014). Is Research Working for You: Self-Assessment Tool.

[53] Belfast Healthy Cities: https://www.belfasthealthycities.com/ Accessed 22–07-17.

[54] Economic and Socail Research Council, Research Ethics: https://esrc.ukri.org/funding/guidance-for-applicants/research-ethics/ Accessed 12–11-17.

[55] Economic and Socail Research Council, Research Data Policy: https://esrc.ukri.org/funding/guidance-for-grant-holders/research-data-policy/ Accessed 27–08-17.

[56] James P, Jankowska M, Marx C, Hart JE, Berrigan D, Kerr J, Hurvitz PM, Hipp JA, Laden F (2016). Spatial energetics. Am J Prev Med.

